# HPV16 E6 Promotes the Progression of HPV Infection-Associated Cervical Cancer by Upregulating Glucose-6-Phosphate Dehydrogenase Expression

**DOI:** 10.3389/fonc.2021.718781

**Published:** 2021-10-07

**Authors:** Ye-Fei Chang, Guo-Ji Yan, Guang-Cai Liu, Ying Hong, Hong-Lan Chen, Shui Jiang, Yong Zhong, Yan-Bin Xiyang, Tao Hu

**Affiliations:** ^1^ Department of Laboratory Medicine, The Third People’s Hospital of Yunnan Province, Kunming, China; ^2^ Institute of Neuroscience, Basic Medical College, Kunming Medical University, Kunming, China

**Keywords:** E6 protein, human papillomavirus, glucose-6-phosphate dehydrogenase, cervical cancer, HPV16 E6

## Abstract

Cervical cancer, which is significantly associated with high-risk human papillomavirus (HPV) infection, currently ranks the fourth most common cancer among women worldwide. Previous literature reported that the elevated expression of G6PD was significantly correlated with the occurrence and deterioration of human cervical cancer, especially with the cervical cancer with HPV16 and HPV18 infection. In this study, we verified that G6PD expression has a strong positive correlation with HPV16 E6 levels in cervical cancer tissues and cells. In addition, regulating the expression of HPV16 E6 significantly affected the proliferation, apoptosis, migration, and invasion in the cervical cancer HeLa cells, as well as the transcript and protein levels of G6PD. The luciferase reporter assay and ChIP assay proved that HPV16 E6 stimulated the transcription of G6PD mRNA and subsequently enhanced the expression of G6PD through directly binding to the specific sites in the promoter of G6PD. Our findings reveal that HPV16 E6 is a novel regulatory factor of G6PD. Furthermore, by regulating the expression of G6PD, HPV16 E6 might promote the proliferation and migration potential, and inhibit apoptosis of cervical cancer cells, which ultimately contributed to the progression and metastasis of cervical cancer.

## Introduction

Cervical cancer is a common cancer in women, which often originates in the lining of the cervix, usually at the junction of the squamous and columnar epithelium (1). According to the global survey statistics in 2018, cervical cancer has become the fourth most common cancer among women, and accounts for 7.5% of all female cancer deaths. In 2018 statistics alone, more than 300,000 female patients died of cervical cancer (2, 3). At present, although more effective screening methods and vaccine preventive measures have been developed, there are still a large number of women in the world threatened by this disease. In view of its higher incidence and greater harm in human females, cervical cancer has become a global health priority, and researchers are making unremitting efforts to explore its pathogenesis and to seek potential measures for prevention and therapy.

It is well known that human papillomavirus (HPV), as the most common cause of cervical cancer in women, is a circular non-enveloped double-stranded DNA virus. It is one of the necessary factors for precancerous lesions and invasive cancer of female reproductive tract (4). Up to now, researchers have discovered more than 40 HPV gene types that have the ability to infect human reproductive organs, and 15 of them have been found to have carcinogenic effects (3). According to the statistics from Elaine M Smith and Ana Cecilia Rodríguez, 70% of all invasive cervical cancer is related to gene types 16 and 18, which account for about 50% of all cervical cancers with HPV infection (5, 6).

HPV16, which belongs to a high-risk type, is closely related to invasive cervical cancer. It is well established that the expression of HPV16 E6 is necessary for transformation from normal cells into malignant cells and maintenance the malignant features. Furthermore, the HPV oncogene E6 is proved to induce functional suppression of the tumor suppressor gene p53, by integrating into the host genome (7, 8). Instead of directly binding to p53, E6 protein inhibits p53 function through alternative ways, such as by binding to E3 ubiquitin ligase and E6 associated protein (E6AP), and subsequently forming a ternary complex with p53 to make it ubiquitin (1). The inactivation and degradation of p53 reduce the transcriptions of the Notch1 gene, which serves as a cancer suppressor gene in cervical keratinocytes (9). Moreover, through interfering the physiological functions of GADD34/PP1, Procaspase 8, FADD, or Bak, which are involved in apoptosis inhibition in tumor progress, E6 induces disturbance in the apoptosis procedure during tumor formation and development (10–13). Therefore, E6 diminishes even impairs the tumor inhibition effects of tumor suppressors and lead to the occurrence and progress of cervical cancer (9).

Glucose-6-phosphate dehydrogenase (G6PD) is one of the rate-limiting enzymes in the pentose phosphate pathway. G6PD has the ability to produce ribose through the pentose phosphate pathway. During the oxidation reaction, one of the functions of G6PD is to produce NADPH; thus, G6PD plays an important role in maintaining redox homeostasis in oxidative stress (14). Current studies reveal that the increases in G6PD expression and activity are detected in a variety of tumors (15–18). Our previous study reports that the elevated G6PD in patients with HPV16 or HPV18 infection, while inhibiting G6PD, induces significant reduction in the proliferation and increase in apoptosis of both HPV16+ Siha and HPV18+ HeLa cells (19, 20). Previous results further proved the positive correlation between the ectopic expression of G6PD and high-risk HPV16- and HPV18-infected cervical cancer (19, 20). Others also observe an abnormal increase in G6PD activity in cervical intraepithelial neoplasia (CIN) tissues (21, 22), which coincides with our results.

Considering those, there has been an international consensus that G6PD is a novel biomarker of diagnosis and prognosis and may be used as a potential candidate for cervical cancer treatment. However, the exact mechanisms underlying the abnormal expression of G6PD and the possible effects between HPV E6 and G6PD in the progress and deterioration of high-risk HPV16 infected cervical cancer are still an unsolved mystery. We propose that HPV16 E6 leads to the progression and metastasis of cervical cancer by regulating the expression of host G6PD.

## Materials and Methods

### Preparation of Human Samples

A total of 57 tumor samples of cervical cancer patients who were treated and operated in the Third people’s Hospital of Yunnan Province from July 2016 to August 2019 were collected. Samples from 15 patients diagnosed with cervical prolapse served as non-cancer control. All the patients provided informed consent for their participation in the study. Forty-eight cases from the 57 patients described above were included in this study. Among these patients, 35 women were less than 50 years old and 13 were older than 50 years old. All 48 patients were HPV16 DNA-positive, which were identified by PCR. The exclusion criteria of patients were described as follows: without HPV16 infection, accepting other therapy before visiting the clinic of our hospital, other organic or systemic diseases, recurrent infections, and HIV-infected and other immunosuppression conditions. The samples were collected and were independently identified by three experienced pathologists. According to pathological status, the 48 cases were identified as cervical squamous cell carcinoma. The clinicopathological characteristics of these patients were described in **Table 1**.

**Table 1:** Clinicopathological characteristics of patients with cervical cancer.

Characteristic	No. of patients	% (*n* = 48)
**Age**		
≤50	35	73
>50	13	27
**FIGO stage**		
I	8	17
II	19	40
III	17	35
IV	4	8
**Histological grade**		
G1	11	23
G2	28	58
G3	9	19
**Tumor size**		
≤4 cm	29	60
>4 cm	19	40
**Lymph node metastasis**		
Negative	44	92
Positive	4	8
**Lymphovascular invasion**		
No	46	96
Yes	2	4

### Plasmids and siRNA Preparation

The full-length coding region of HPV16 E6 was amplified by RT-PCR and then cloned into pcDNA3.1 vector between the recognition sequence for *Hind*III and *Xho*I. Briefly, reverse transcription–polymerase chain reaction (RT-PCR) was conducted to amplify the cDNA clones of the full-length coding region of HPV16 E6, which concluded the restriction sites for *Hind*III and *Xho*I enzymes, using the PCR Master Mix kit (Invitrogen; Thermo Fisher Scientific, Inc.). The primer sequences used in this study are shown in **Table 2**. Afterwards, the coding region of E6 sequences was digested with *Hind*III and *Xho*I restriction enzymes and ligated to pcDNA3.1 plasmid (Thermo Fisher Scientific, Inc.). E6 expression vector (pcDNA E6) was transfected into cells by Lipofectamine 3000 (Thermo Fisher Scientific, Inc.) essentially as described by the manufacturer’s instruction, and the pcDNA3.1 vector was used as control (vector control). A similar process for overexpression of G6PD in HeLa cells was performed as described previously (20). The plasmids psPAX2 (Addgene) loading the open-reading frames of G6PD were used to upregulate G6PD and named as plenti G6PD, with an empty plasmid as the negative control (G6PD scrambled-control).

**Table 2:** Primer sequences used in RT-PCR assays.

Gene	Primer sequences
HPV16 E6	Forward: 5’-GATCCAACACGGCGACCCTACAAC-3’
	Reverse: 5’-CTGGATTCAACGGTTTCTGGT-3’
GAPDH	Forward: 5’-CGAGATCCCTCCAAAATCAA-3’
	Reverse: 5’-TTCACACCCATGACGAACAT-3’
G6PD	Forward: 5’-TGAGCCAGATAGGCTGGAA-3’
	Reverse: 5’-TAACGCAGGCGATGTTGTC-3’

Specific small interfering RNA (siRNA) aimed at human HPV E6 (siRNA E6; Santa Cruz Biotechnology; SCBT; sc-156008) and G6PD (G6PD siRNA; Santa Cruz Biotechnology; sc-60667), as well as the scrambled sequence control (Empty-siRNA E6; G6PD siRNA NC, respectively), were designed and purchased from Santa Cruz Biotechnology Inc. For each experiment, 35 nM of pooled siRNA was used and cell lysates were harvested after 48 h.

### Cell Culture and Treatment

The HeLa cells used in this study were purchased from the Institute of Biochemistry and Cell Biology, Shanghai, China. Cells were cultured in Dulbecco’s modified Eagle medium (DMEM, Gibco, Life Technologies, Carlsbad, CA) containing 10% fetal bovine serum and 1% penicillin/streptomycin, and incubated at 37°C with 5% CO_2_.

To determine whether HPV E6 contributed to carcinogenic events in high-risk HPV-infected cervical cancer by regulating G6PD, HPV E6 overexpression and/or inhibition was established in cultured cervical cancer cells with or without G6PD expression regulation. HeLa cells were transfected with the pcDNA E6, pcDNA vector, plenti G6PD, G6PD scrambled-control, G6PD siRNA, and/or G6PD siRNA NC. In details, HeLa cells were co-transfected with pcDNA E6 along with the G6PD siRNA (named E6 + G6PD siRNA). In control cells, G6PD siRNA was replaced by G6PD siRNA negative control (named E6 + G6PD siRNA NC). Cultured HeLa cells were also transfected combined with pcDNA E6 and plenti G6PD (named E6 + plenti G6PD) or G6PD scrambled vector as a control (named E6 + G6PD scrambled-control). Additionally, HeLa cells with siRNA E6 was co-transfected with plenti-G6PD (named siRNA E6 + plenti-G6PD) or Empty-siRNA served as a control (named siRNA E6 + G6PD control). Similarly, HeLa holding siRNA E6 was co-transfected with G6PD siRNA; co-transfection with Empty-siRNA served as a control (named siRNA E6 + G6PD siRNA and siRNA E6 + G6PD siRNA NC, respectively).

After 48 h of transfection, the transfected cells were harvested and subjected to the following assays. RT-qPCR and Western blot were performed to validate the efficiency of transfection with various treatments. Proliferation, apoptosis, migration, and invading were then examined in these cells using 3-(4,5-dimethylthiazol-2-yl)-2,5-diphenyltetrazolium bromide (MTT), flow cytometry (FCM) assays, and transwell assays, respectively. The HeLa cells loading pcDNA E6 were also employed to perform luciferase reporter assay and chromatin immunoprecipitation (ChIP) assay.

### Cell Proliferation Assay

In this experiment, MTT assay was used to determine the changes of cell viability following different treatments. Firstly, the cells were inoculated with 96-well culture plates, and the cell inoculation density was set at 5 × 10^3^ cells/ml per well, and the medium was 200 ml. The incubation time was set at 24–96 h. After the set incubation time, 20 ml of 5 mg/ml MTT reagent was added to each well and incubated at 37°C for 4 h. After incubation, the medium was sucked out and 150 ml of dimethyl sulfoxide (DMSO) reagent was added to each well. The optical density of each tested sample was immediately determined using a microplate reader (Biorad, Hercules, CA), and the determination benchmark was set at 490 nm.

### FCM Assay

As previously described, Annexin V-FITC-flow cytometry assay kit (4A Biotech Co. Ltd.) was used to evaluate apoptosis rates in HeLa cells with overexpressed or siRNA interfered HPV16 E6 after introduction with G6PD overexpression plasmids or specific siRNA (23).

### Transwell Assays

As previously described (24, 25), HeLa cells with various treatments were cultured in a six-well plate until the cells were completely fused. The cells were inoculated on the Modified Boyden Chambers in 24-well Tissue Culture Plates (BD Biosciences, San Jose, CA, USA) in the appropriate number. Six hundred microliters of DMEM medium with 10% FBS was added into the bottom compartment. After incubation for 24 h, 4% polymethanol was fixed and then stained with 0.1% crystal violet. The stained cells that migrated or invaded the dorsal side of the compartment were photographed and counted under an IBX3 inverted microscope (Olympus Corporation) under 200× magnification. Five regions were randomly selected in each group to count the number of migrated or invaded cells.

### Reverse Transcription–Quantitative Polymerase Chain Reaction Analysis

RT−qPCR was used to quantify the mRNA expression levels of G6PD. All the primers employed in the present study were synthesized by Invitrogen; Thermo Fisher Scientific, Inc. The tumor samples and cultured HeLa cells were additionally obtained for G6PD detection, as previously described (20). TRIzol^®^ reagent (Invitrogen; Thermo Fisher Scientific, Inc.) was used to extract the total RNA, according to the manufacturer’s protocol. The first−strand cDNA was synthesized from 2 µg of total RNA, using Revert Aid™ First Strand cDNA Synthesis kit (Takara Biotechnology Co., Ltd.). PCR was subsequently conducted using the 2× Mix SYBR-Green I (Beijing Biosea Biotechnology Co., Ltd., Beijing, China; 10 µl), primer (0.25 µl; 10 pmol/l), template DNA (1 µl), and sterile water (8.5 µl). qPCR amplification was performed at 95°C for 3 min, followed by 37 thermocycling steps consisting of denaturation at 94°C for 30 s, annealing at 57°C for 35 s, and extension at 72°C for 30 s. The relative quantification cycle (Cq) method was used to compare target gene expression between samples. Fold changes in target gene expression were determined relative to a blank control following normalization to the GAPDH house keeping gene using the 2^−ΔΔCq^ method (26).

### Western Blot

Western blot was used to detect the expression of E6 protein and/or G6PD protein in tumor tissues or HeLa cells with different treatments as described previously (20). Briefly, cells or samples were lysed on ice for 30 min in CytoBuster Protein Extraction Buffer (Novagen, USA) and then all samples were centrifuged at 12,000 rpm at 4°C. Quantitative 50-g proteins of each sample was extracted and isolated by 10% sodium dodecyl sulfate polyacrylamide gel electrophoresis (SDS-PAGE). Then, the protein was transferred to a nitrocellulose (NC) membrane and sealed with Tris-Buffered Saline Tween-20 (TBST) containing 5% non-fat milk. The membrane was subsequently incubated with mouse anti-human HPV E6 antibody (1:800, Merck Millipore, MAB874, USA), goat anti-human G6PD proteins (1:500, Santa Cruz, sc-46971) or mouse anti-human GAPDH (1:500, Santa Cruz, sc-81545) antibodies at 4°C overnight. After washing in TBST, the membrane was incubated with horseradish peroxidase (HRP)-conjugated secondary antibodies (1:2,000) at 25°C for 4 h. The protein was visualized and the quantity of targeting protein was determined using electrochemiluminescence (ECL) technique (BestBio, USA). All results were photographed using the Bio-Rad Gel Imagining system (ChemiDoc™XRS+) and using Quantity One software v4.6.6 (all Bio-Rad Laboratories, Inc., Hercules, CA, USA) to calculate the gray density.

### Luciferase Reporter Assay

The sequence contains the 5’-flanking region of the human G6PD promoter (from +12 to 817 bp) and was amplified by PCR, and the amplified product was inserted into the pGL3 plasmid vector (Thermo Fisher Scientific, Inc.) and linked to the luciferase reporter gene (pGL3-G6PD-Luc). Afterwards, the HeLa cells were transfected with pGL3-G6PD-Luc and Renilla luciferase plasmids (internal control) were used as the vector control, or the recombinant HPV16 E6 protein (His tag; Abcam, ab226447). The changes in luciferase activity were observed and recorded. If E6 binds to the G6PD promoter and activates the transcription of G6PD, the luciferase activity will be significantly increased compared with the control group. The data represent at least three independent experiments. Triplicate samples were analyzed in each experiment.

### ChIP Assay

The ChIP kit (Pierce Chemical, Dallas, USA) was employed and ChIP analysis was performed according to the protocols provided by the manufacturer. The anti-HPV16 E6 antibody (Santa Cruz Biotechnology; CIP5, sc-460) and mouse immunoglobulin-G (IgG; Merckmillipore, 12-371B) were employed. RT-qPCR was performed to quantify the enrichment of DNA fragments in immunoprecipitated HeLa cells. The primers were designed according to the putative promoter regions of G6PD.

### Statistical Analysis

Statistical analysis of experimental data was performed by SPSS21.0 version. Data are presented as means ± standard deviation (SD). In the enumeration data, the data with homogeneity of variance after homogeneity test were analyzed by LSD and SNK tests by one-way ANOVA, while the data with heterogeneity of variance were analyzed by Tamhane ‘s T2 and Dunnett ‘s T3 tests. When *p*-value < 0.05, the difference between relevant data was considered to be statistically significant.

## Results

### Expression and Correlation Analysis of G6PD and HPV16 E6 Protein in Patients With Cervical Cancer

**Figure 1:**
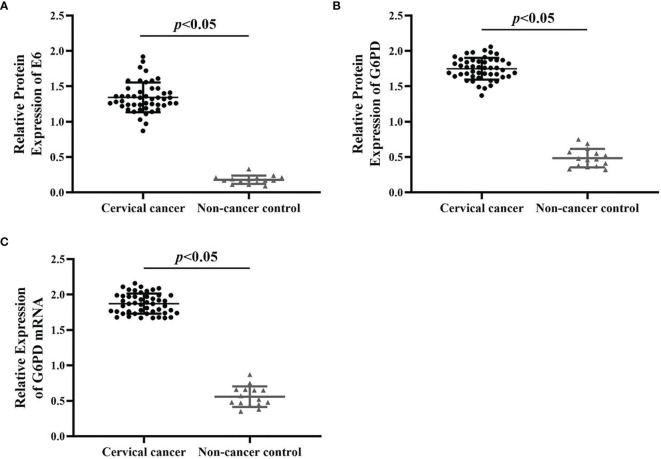
The expression changes of HPV E6 and G6PD between the cervical cancer cases and normal control. The relative expression levels of E6 protein **(A)**, G6PD protein **(B)**, and G6PD mRNA **(C)** in cervical cancer patients (*n* = 48) and non-cancer control ones (*n* = 15) were shown in the scatter dot plots. The scatter points represented the original data of individuals within each group. The quantitative analysis of expression levels of E6 and G6PD showed statistical differences. Data are presented as means ± standard deviation (SD).

The acquisition methods and inclusion criteria of human tissues used in this study were described in the *Materials and Methods* section. The tumor tissues of patients with different pathological stages were detected, mainly for the expressions of HPV16 E6 (**Figure 1A**) and G6PD protein (**Figure 1B**)/mRNA (**Figure 1C**), and the correlation between them was analyzed.

As shown in **Figure 1**, by using qRT-PCR and Western blot analysis, the results revealed that the expression levels of E6/G6PD protein (**Figures 1A, B**) and G6PD mRNA (**Figure 1C**) in cervical cancer groups were significantly higher than that of non-cancer control groups [HPV E6 protein: degree of freedom (df) = 62, *F* = 441.372, *p* = 0.000; G6PD protein: df = 62, *F* = 821.223, *p* = 0.000; G6PD mRNA: df = 62, *F* = 968.337, *p* = 0.000]. Furthermore, the expression level of HPV16 E6 was positively correlated with the expression levels of G6PD in host tumor tissues (correlation analysis, *r* = 0.89, *p* < 0.05).

### HPV16 E6 Affects the Expression of G6PD in HeLa Cells

**Figure 2:**
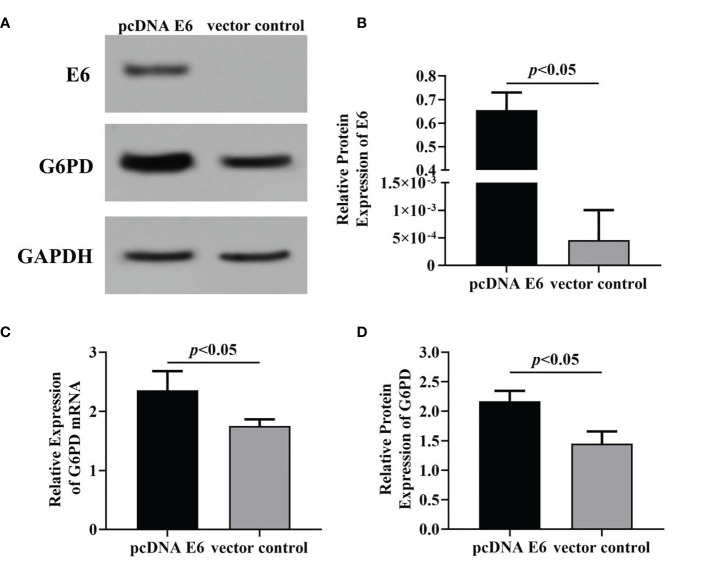
The effects of HPV16 E6 on G6PD expression. Forty-eight hours after plasmid transfection, the levels of E6 protein and G6PD mRNA/protein in HeLa cells were detected. The expression levels of E6 protein **(A, B)** and G6PD mRNA/protein **(A, C, D)** were indicated by Western blots or RT-qPCR, respectively. **(A)**, representative blots from two groups. Statistical plots of the relative expression of E6 protein **(B)**, G6PD mRNA **(C)**, and protein **(D)**. Data are presented as means ± SD (*n* = 5).

In this study, the plasmid transfection technology was used to alter the expression of HPV E6 and/or G6PD in HeLa cells. In order to establish cells with HPV16 E6 overexpressing, the plasmids pcDNA-HPV16 E6 was transfected HeLa cells, and then the HPV16 E6 and G6PD expression were detected 48 h after plasmid transfection. The results of mRNA/protein levels of E6 and G6PD indicated by RT-qPCR and Western blot proved that the transfection is effective (**Figure 2A**). Overexpression of HPV16 E6 significantly increased the expression level of either transcription or protein of G6PD, when compared with that of the vector-treated control group (HPV E6 protein: *p* = 0.008; G6PD protein: df = 9, *F* = 35.463, *p* = 0.000; G6PD mRNA: df = 9, *F* = 15.031, *p* = 0.005; **Figures 2B–D**).

**Figure 3:**
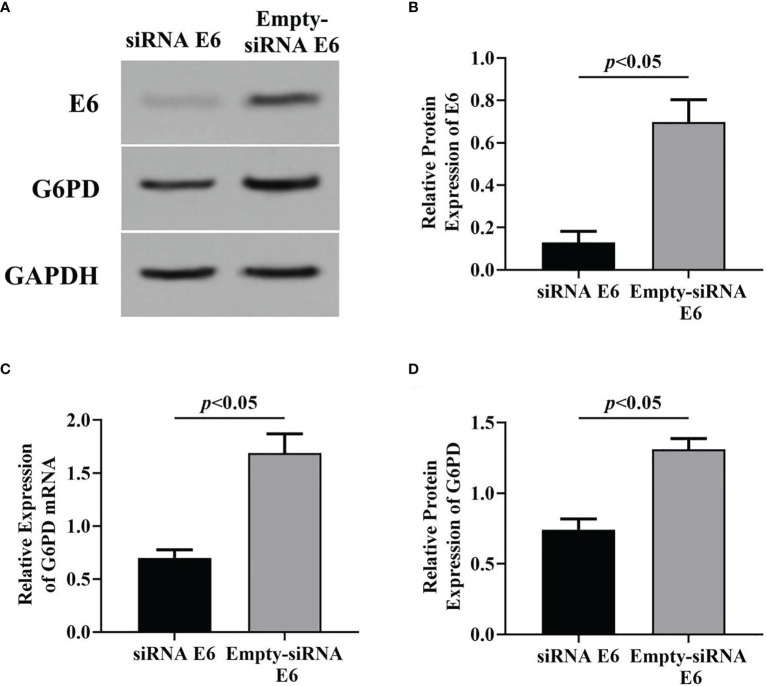
Effects of inhibition of HPV16 E6 expression on G6PD expression. After stable expression of pcDNA HPV16 E6 plasmid in HeLa cells, siRNA E6 was employed to interfere its expression. Then, the relative expression of E6 protein and G6PD mRNA/protein was detected. The expression levels of E6 protein **(A, B)** and G6PD mRNA/protein **(A, C, D)** were also indicated by Western blots or RT-qPCR. **(A)** Representative blots from two groups. Statistical plots of the relative expression of E6 protein **(B)**, and G6PD mRNA **(C)** and protein **(D)**. Data are presented as means ± SD (*n* = 5).

Subsequently, the specific siRNA targeting HPV E6 was employed to interfere with the expression of HPV16 E6 in HeLa cells loading pcDNA E6 plasmids. Results of Western blot (**Figures 3A, B, D**) and RT-qPCR (**Figure 3C**) showed that following siRNA E6 treatment, the expression levels of HPV E6 and G6PD significantly decreased, compared with that of the empty-siRNA E6 control group (HPV E6 protein: df = 9, *F* = 115.885, *p* = 0.000; G6PD protein: df = 9, *F* = 139.423, *p* = 0.000; G6PD mRNA: df = 9, *F* = 139.987, *p* = 0.000; **Figures 3B–D**). These data proved the siRNA E6 transfection was effective, and the HPV E6 regulated the G6PD expression in HeLa cells.

### Validation of Plenti-G6PD and G6PD siRNA Treatments

To determine the transfection efficiency of overexpression and interfering fragments targeting G6PD, the expression levels of G6PD mRNA (indicated by RT-qPCR) and protein (indicated by Western blot) were evaluated following the plasmids plenti-G6PD or G6PD siRNA transfection in HeLa cells.

**Figure 4:**
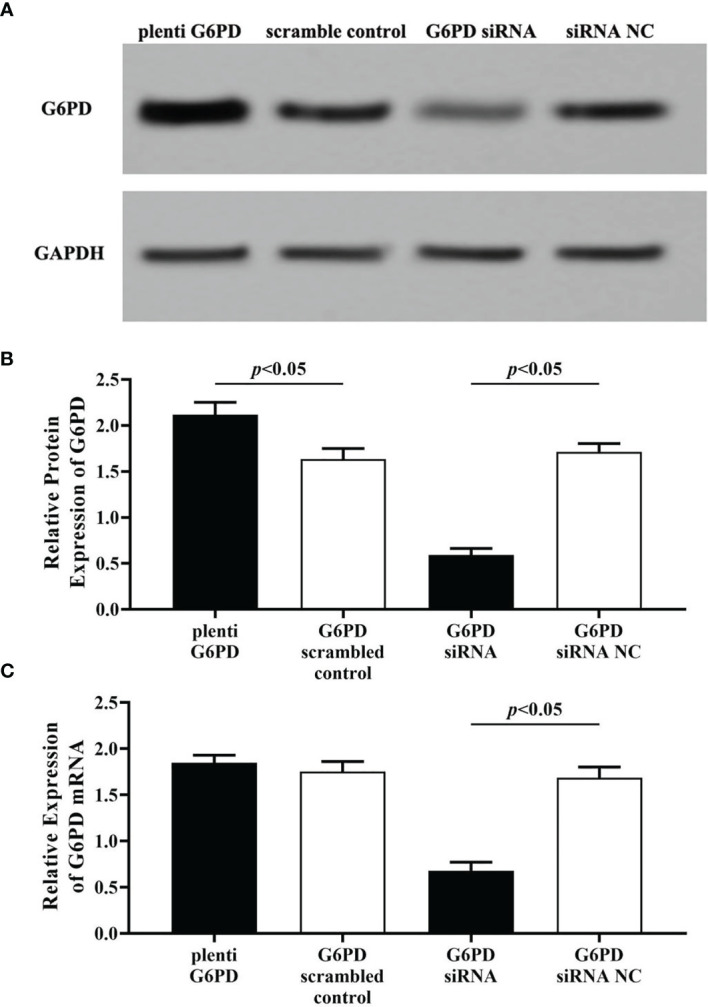
Evaluation the transfection efficiency of plenti G6PD and G6PD siRNA. The expression levels of G6PD protein **(A, B)** and G6PD mRNA **(C)** were indicated by Western blots or RT-qPCR. **(A)** Representative blots from four groups. Statistical plots of the relative expression of G6PD protein **(B)** and G6PD mRNA **(C)**. Data are presented as means ± SD (n = 5).

Compared with the scrambled control group, plenti-G6PD transfection induced marked increase in G6PD protein levels (df = 9, *F* = 37.302, *p* = 0.000, *p* < 0.05, **Figures 4A, B**). However, plenti G6PD transfection showed no significant effects on the transcript levels of G6PD mRNA (df = 9, *F* = 2.439, *p* = 0.157, *p* > 0.05, **Figure 4C**). By contrast, treatment with G6PD siRNA effectively reduced the endogenic expression levels of G6PD, including G6PD protein and G6PD mRNA [compared with the matched siRNA negative control (NC), G6PD protein: df = 9, *F* = 484.186, *p* = 0.000; G6PD mRNA: df = 9, *F* = 239.412, *p* = 0.000, *p* < 0.05, **Figures 4B, C**].

Statistical results revealed that G6PD overexpression or interfering fragments, which named plenti G6PD and G6PD siRNA, induced corresponding increase or decrease in G6PD levels, which suggested that plenti G6PD or G6PD siRNA employed in this study were valid in HeLa cells.

Plenti G6PD, HeLa cells were transfected with plenti G6PD; G6PD scrambled-control, HeLa cells were transfected with G6PD scrambled vector; G6PD siRNA, HeLa cells were transfected with G6PD siRNA; G6PD siRNA NC, HeLa cells were transfected with G6PD siRNA negative control.

### The Relationship Between the Expression of E6 and G6PD

In order to explore whether HPV E6 takes part in the carcinogenic progress of cervical cancer by regulating G6PD, the G6PD expressional upregulation (plenti G6PD) or downregulation (G6PD siRNA) were conducted in HeLa cells on the basis of the HPV16 E6 siRNA and/or overexpressing, respectively. Afterwards, the expression levels of E6 and G6PD were assessed by RT-qPCR and/or Western blot.

**Figure 5:**
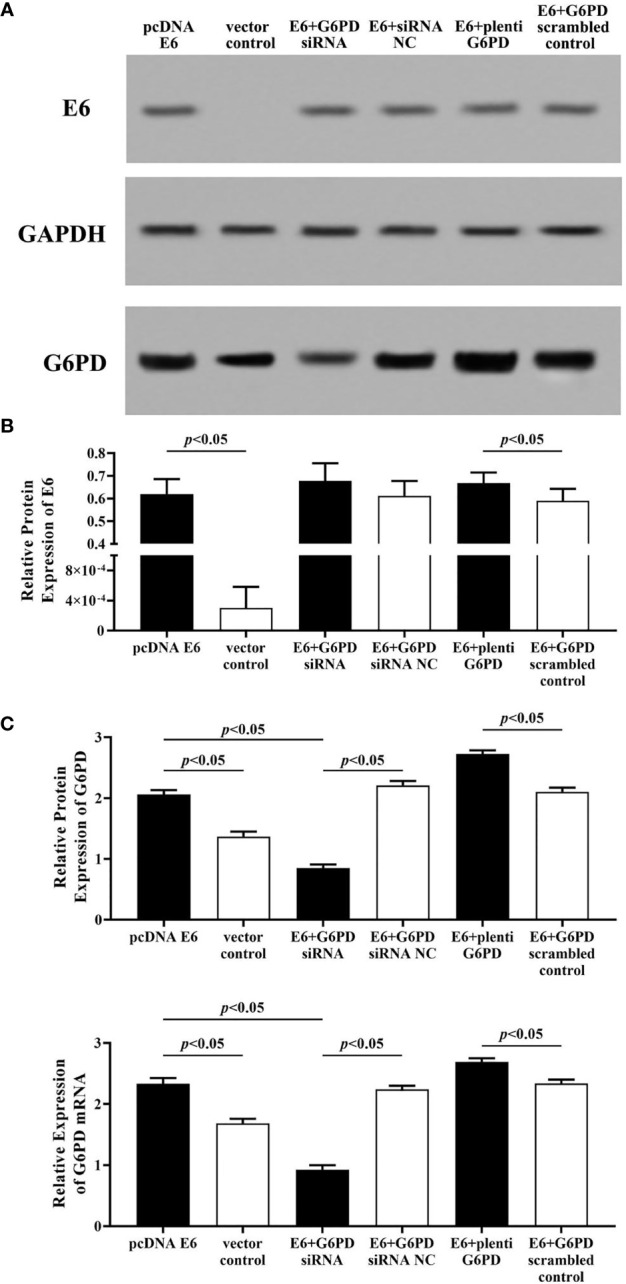
HPV E6 expressional changes regulate the transcriptions and protein levels of G6PD in HeLa cells. The expression levels of E6 protein **(A, B)** and G6PD protein **(A, C)** /mRNA **(C)** were indicated by Western blots and/or RT-qPCR. **(A)** Representative blots from six groups. Statistical plots of the relative expression of E6 protein **(B)**, and G6PD protein/mRNA **(C)**. Data are presented as means ± SD (n = 5).

As shown in **Figure 5**, after treatment with the plasmids pcDNA-HPV16 E6 in the HeLa cells, the expression levels of E6 protein and G6PD protein/mRNA increased significantly (HPV E6 protein: df = 29, *F* = 104.006, *p* = 0.000; G6PD protein: df = 29, *F* = 450.131, *p* = 0.000; G6PD mRNA: df = 29, *F* = 388.997, *p* = 0.000, *p* < 0.05; **Figures 5A–C**). Combined application of pcDNA HPV16 E6 and G6PD siRNA showed no statistical effect on E6 expression, but induced further decrease in the expression of G6PD, compared with the E6+G6PD siRNA NC control group and the pcDNA-HPV16 E6 transfected group, respectively (HPV E6 protein: df = 29, *F* = 104.006, *p* = 0.080; G6PD protein: df = 29, *F* = 450.131, *p* = 0.000; G6PD mRNA: df = 29, *F* = 388.997, *p* = 0.000, *p* < 0.05; **Figures 5A–C**). Furthermore, combination with pcDNA-HPV16 E6 and plenti G6PD led to a significant increase in the expression levels of both E6 and G6PD, compared with their control groups, respectively (HPV E6 protein: df = 29, *F* = 104.006, *p* = 0.041; G6PD protein: df = 29, *F* = 450.131, *p* = 0.000; G6PD mRNA: df = 29, *F* = 388.997, *p* = 0.000, *p* < 0.05; **Figures 5A–C**).

**Figure 6:**
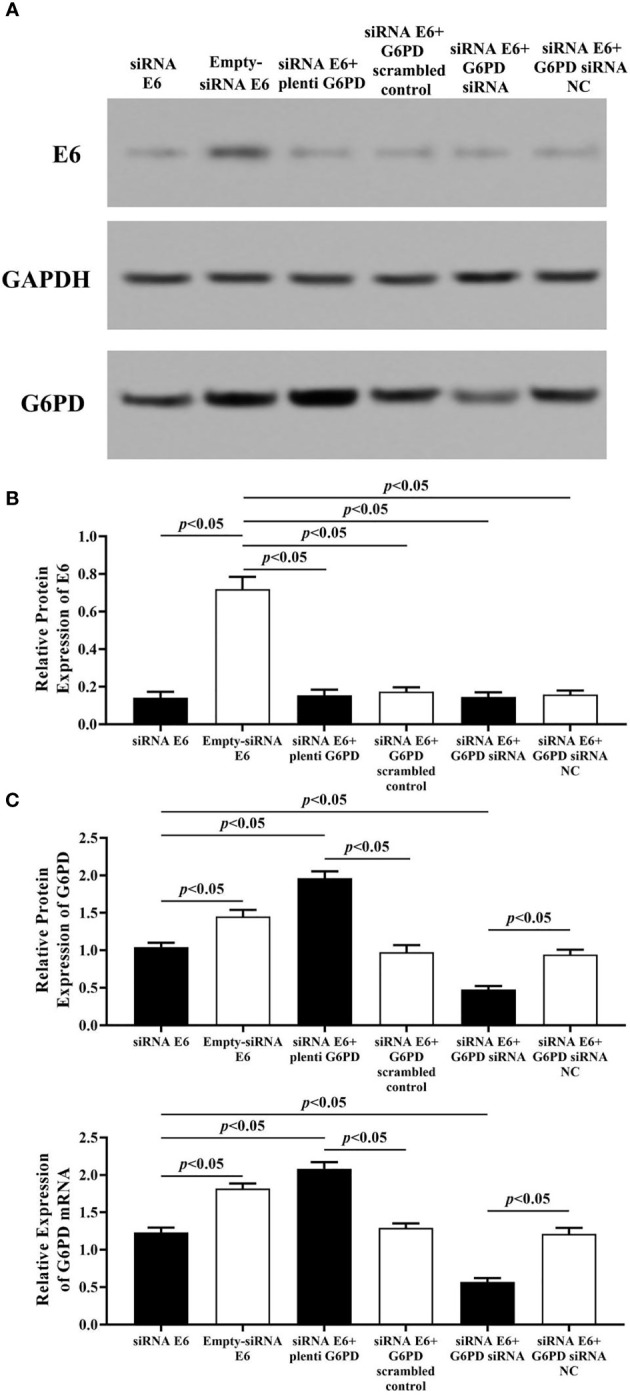
HPV E6 expressional changes regulate the transcriptions and protein levels of G6PD in HeLa cells. The expression levels of E6 protein **(A, B)** and G6PD **(A, C)**/mRNA **(C)** were indicated by Western blots or RT-qPCR. **(A)** Representative blots from six groups. Statistical plots of the relative expression of E6 protein **(B)**, and G6PD protein/mRNA **(C)**. Data are presented as means ± SD (n = 5).

Employment of HPV16 E6 siRNA significantly induced reduction in the expression levels of E6 and G6PD protein/mRNA, compared with the Empty-siRNA E6 control ones (HPV E6 protein: df = 29, *F* = 207.113, *p* = 0.000; G6PD protein: df = 29, *F* = 218.261, *p* = 0.000; G6PD mRNA: df = 29, *F* = 285.205, *p* = 0.000, *p* < 0.05; **Figures 6A–C**). In HeLa cells co-transfected with siRNA E6 and G6PD siRNA, the expression levels of G6PD protein/mRNA were statistically decreased compared to the control groups (G6PD protein: df = 29, *F* = 218.261, *p* = 0.000; G6PD mRNA: df = 29, *F* = 285.205, *p* = 0.000, *p* < 0.05; **Figure 6C**). However, there was no significant difference in the expression of E6 protein between groups that co-transfected with siRNA E6+G6PD siRNA and siRNA E6+G6PD siRNA NC (df = 29, *F* = 207.113, *p* = 0.602, *p* > 0.05; **Figure 6B**). To further verify and supplement our point of view that HPV16 E6 might have the ability to regulate the expression of G6PD, HeLa cells with siRNA E6 were co-transfected with plenti G6PD. The expression level of E6 protein between the siRNA E6+plenti G6PD group and its control group had no statistical difference (df = 29, *F* = 207.113, *p* = 0.387, *p* > 0.05; **Figure 6B**). Moreover, the expression levels of G6PD protein as well as G6PD mRNA were significantly increased (G6PD protein: df = 29, *F* = 218.261, *p* = 0.000; G6PD mRNA: df = 29, *F* = 285.205, *p* = 0.000, *p* < 0.05, **Figure 6C**).

Therefore, the results above proved a significant correlation between the expression of HPV16 E6 and G6PD, while HPV16 E6 might regulate the mRNA and protein levels of G6PD in HeLa cells.

### HPV16 E6 Promotes Viability, Migration, and Invasion and Inhibits Apoptosis of Cervical Cancer Cells by Regulating G6PD

In this study, the MTT assay was used to evaluate the cell viability, the flow cytometry (FCM) assay was performed to detect the apoptosis rates, and the transwell assay was employed to assess the mobility of HeLa cells with various treatments.

**Figure 7:**
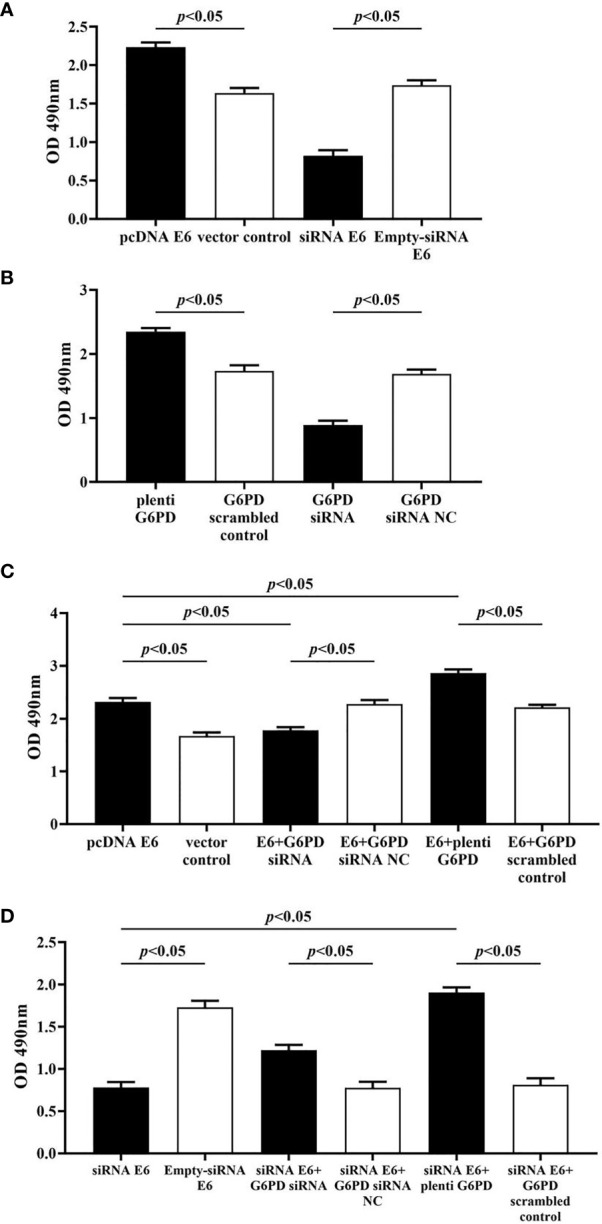
Changes in proliferation activity of HeLa cells with different treatments. The statistical plots of the cell viability changes in groups that transfected with pcDNA E6 or siRNA E6 **(A)** alone, plenti G6PD or G6PD siRNA **(B)** alone, as well as combining these treatments **(C, D)**. Data are presented as means ± SD (n = 5).

**Figure 8:**
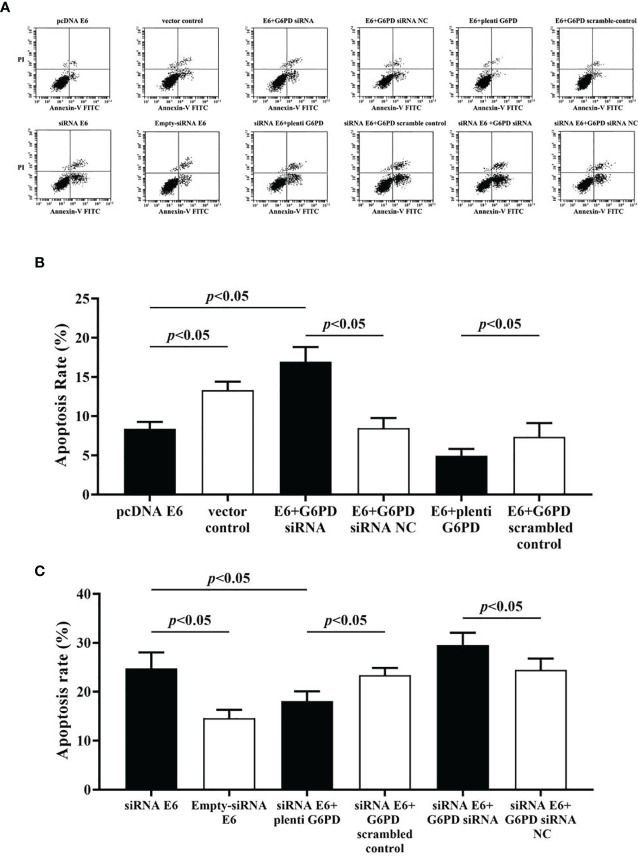
Apoptosis rate of HeLa cells with various treatments were evaluated by the flow cytometry (FCM) assay. **(A)** Representative apoptosis records indicated by Annexin-V FITC-PI assay derived from HeLa cells accepted different treatments. **(B, C)** Statistical plots in apoptosis rates of HeLa cells with different treatments. Data are presented as means ± SD (*n* = 5).

**Figure 9:**
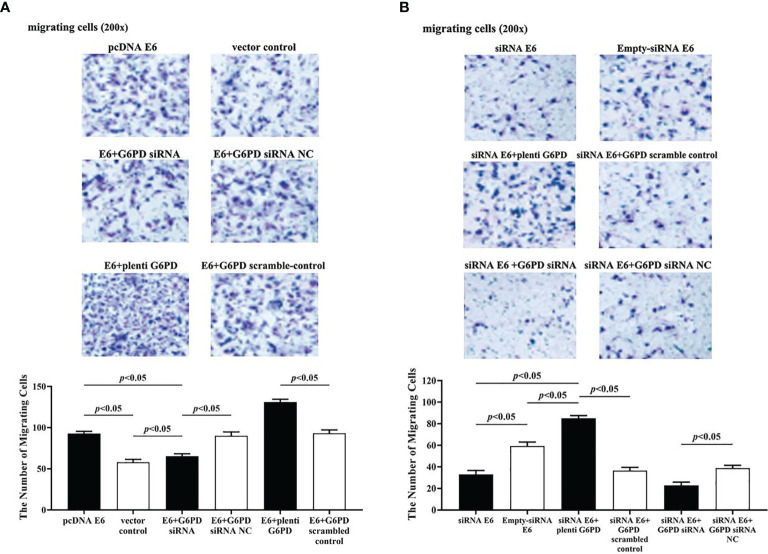
HPV16 E6 promotes migration of HeLa cells by G6PD regulation. The staining of migratory cells with different transfections was observed under a microscope (200×). **(A, B)** Representative photographs and statistical plots of the quantification of migratory cells from six different treatment groups. Data are presented as means ± SD (n = 5).

**Figure 10:**
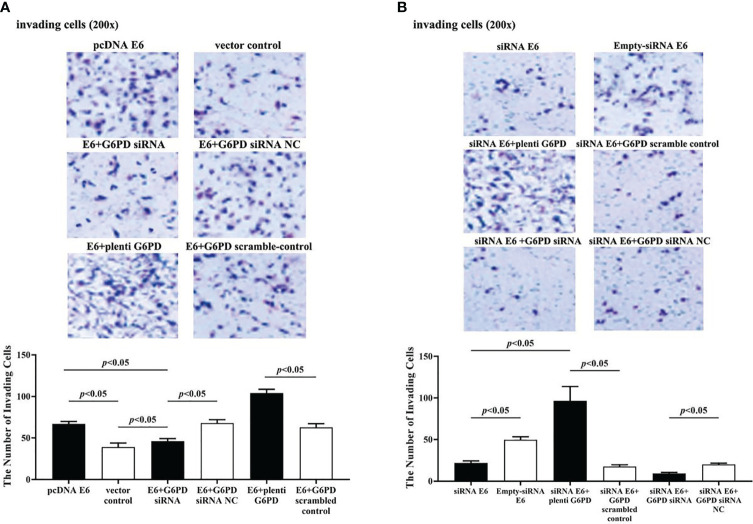
HPV16 E6 facilitates the invasion of HeLa cells through G6PD.The staining of invaded cells with different plasmid transfection was observed under a microscope (200×). **(A, B)** Representative photographs and statistical plots of the quantification of invading cells from six different treatment groups. Data are presented as means ± SD (n = 5).

The statistical results showed that overexpression of E6 induced significant increases in the cell viability (df = 9, *F* = 230.712, *p* = 0.000, *p* < 0.05; **Figure 7A**), decreases in the apoptosis rate (df = 29, *F* = 53.383, *p* = 0.001, *p* < 0.05; **Figures 8A, B**), as well as enhanced cell migration and invasion (cell migration: df = 29, *F* = 252.651, *p* = 0.000; cell invasion: df = 29, *F* = 158.767, *p* = 0.000, *p* < 0.05; **Figures 9A**, **10A**). However, HeLa cells transfected with E6 siRNA showed a dramatic decline in the cell viability (df = 9, *F* = 437.464, *p* = 0.000, *p* < 0.05; **Figure 7A**), significant increase in the apoptosis rate (df = 29, *F* = 27.086, *p* = 0.000, *p* < 0.05; **Figures 8A, C**), as well as marked decrease in the amount of cell migration and invasion (cell migration: df = 29, *F* = 261.331, *p* = 0.000; cell invasion: df = 29, *F* = 99.440, *p* = 0.000, *p* < 0.05; **Figures 9B**, **10B**). Overexpression of G6PD significantly increased cell viability (df = 19, *F* = 358.364, *p* = 0.000, *p* < 0.05; **Figure 7B**), while G6PD expressional interference deceased cell viability, compared with the corresponding control group, respectively (df = 19, *F* = 358.364, *p* = 0.000, *p* < 0.05; **Figure 7B**).

Following treatments with HPV16 E6 overexpression or siRNA interference of HPV16 E6 and G6PD expression knockdown (G6PD siRNA) or G6PD overexpression (plenti G6PD) plasmids in HeLa cells, the cell viability, apoptosis rate, invasion, and migration were evaluated, respectively. As the HeLa cells transfected combined with pcDNA E6 and G6PD siRNA (E6 + G6PD siRNA), the cell viability significantly decreased (df = 29, *F* = 212.884, *p* = 0.000, *p* < 0.05; **Figure 7C**), the apoptosis rate observably increased (df = 29, *F* = 53.383, *p* = 0.001, *p* < 0.05; **Figures 8A, B**), while the amounts of cell migration (df = 29, *F* = 252.651, *p* = 0.000, *p* < 0.05; **Figure 9A**) and invasion (df = 29, *F* = 158.767, *p* = 0.000, *p* < 0.05; **Figure 10A**) statistically decreased, compared with the cells treated with pcDNA E6 alone or treated with both pcDNA E6 and G6PD siRNA NC control. On the contrary, combining with pcDNA E6 and plenti G6PD (E6 + plenti G6PD) in HeLa cells significantly induced increased cell viability (df = 29, *F* = 212.884, *p* = 0.000, *p* < 0.05; **Figure 7C**), decreased apoptosis rate (df = 29, *F* = 53.383, *p* = 0.009, *p* < 0.05; **Figures 8A, B**), as well as augmented migration (df = 29, *F* = 252.651, *p* = 0.000, *p* < 0.05; **Figure 9A**) and invasion (df = 29, *F* = 158.767, *p* = 0.000, *p* < 0.05; **Figure 10A**), when compared with the cells treated with pcDNA E6 alone or treated with both pcDNA E6 and G6PD scrambled control, respectively.

Upon transfection with siRNA E6 with G6PD siRNA (siRNA E6 + G6PD siRNA), the cell viability was significantly increased (df = 29, *F* = 263.210, *p* = 0.000, *p* < 0.05; **Figure 7D**), the apoptosis rate was increased (df = 29, *F* = 27.086, *p* = 0.002, *p* < 0.05; **Figure 8A, C**), and the amounts of cell migration (df = 29, *F* = 261.331, *p* = 0.000, *p* < 0.05; **Figure 9B**) and invasion (df = 29, *F* = 99.440, *p* = 0.032, *p* < 0.05; **Figure 10B**) statistically declined, compared with the cells treated with siRNA E6 alone or treated with both siRNA E6 and G6PD siRNA NC, respectively. When HeLa cells were transfected with siRNA E6 and plenti G6PD (siRNA E6 + plenti G6PD), the cell proliferation was statistically increased (df = 29, *F* = 263.210, *p* = 0.000, *p* < 0.05; **Figure 7D**), the apoptosis rate was significantly decreased (df = 29, *F* = 27.086, *p* = 0.021, *p* < 0.05; **Figures 8A, C**), while the amounts of cell migration (df = 29, *F* = 261.331, *p* = 0.000, *p* < 0.05; **Figure 9B**) and invasion (df = 29, *F* = 99.440, *p* = 0.007, *p* < 0.05; **Figure 10B**) were statistically increased.

All the above results demonstrate that HPV16 E6 participates in the occurrence and development of cervical cancer by regulating the expression of host’s G6PD.

### The HPV16 E6 Is Involved in Transcriptional Activation of G6PD

In this study, luciferase reporter assay and RT-qPCR were performed to verify the regulatory effect of HPV16 E6 on G6PD.

**Figure 11:**
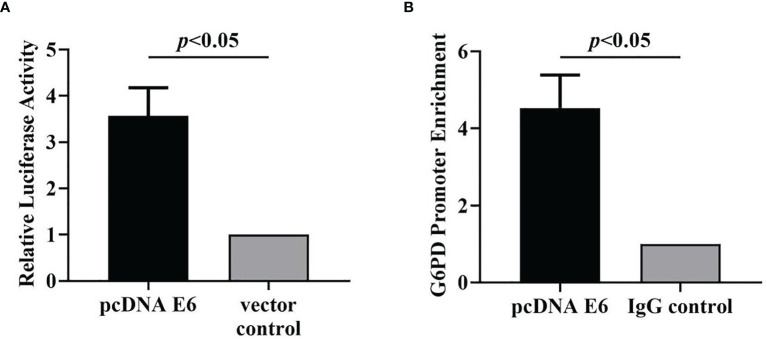
HPV16 E6 activates G6PD transcription and binds directly to the G6PD promoter. The luciferase activity **(A)** and the enrichment of G6PD promoter **(B)** in HeLa cells statistically increased after pcDNA E6 treatment, compared with the control groups, respectively. Data are presented as means ± SD (n = 3).

The results showed that after HPV16 E6 treatment, the luciferase activity increased by 3.6 times compared to the control group, indicating that HPV16 E6 activated the transcription of G6PD (df = 5, *F* = 52.938, *p* = 0.002, *p* < 0.05; **Figure 11A**).

### HPV16 E6 Directly Binds to the Promoter of G6PD

ChIP Assay was used to test whether HPV16 E6 directly binds to the G6PD promoter.

Specifically, the target gene was extracted and amplified by PCR with the primers of specific G6PD promoter, and the enrichment of HPV E6 on G6PD promoter in PCR products from HeLa cells treated with HPV16 E6 antibody was detected. By comparing those two groups, the enrichment of G6PD promoter was significantly increased in the HPV16 E6 treatment group, which was 4.5 times higher than that in the control group (df = 5, *F* = 51.779, *p* = 0.002, *p* < 0.01; **Figure 11B**). The data show that HPV16 E6 directly binds to the G6PD promoter.

## Discussion

Cervical cancer, an important public health and hygiene problem, seriously affects the health of middle-aged to elderly women. One of the crucial reasons for the high impact of the disease is that cervical cancer has a long-term pre-invasive stage before a definite diagnosis. Although histopathological examination may make the diagnosis in the early course of the cancer, E6 and E7 have the effect of inactivating the IFN regulatory factor (IRF), which allows the HPV virus to remain in a long, asymptomatic state of continuous infection (27, 28). This leads to a common phenomenon that the initial symptoms of cervical cancer are often not observed. Currently, cervical cancer is often detected through screening or during physical examination, resulting in a large number of women who are not diagnosed early enough and often have advanced to more serious carcinogenic stages of cancer, which is responsible to the high mortality rate of patients with cervical cancer (3, 29). Therefore, further studies to illustrate the carcinogenic mechanism underlying is necessary, which may provide novel targets for diagnosis at early stage in patients with cervical cancer.

Our research found that the expression levels of E6 protein and G6PD in cervical cancer were significantly increased compared with the non-cancer cohort. Furthermore, the correlation between changes in HPV16 E6 expression and the corresponding alteration in G6PD expression led us to hypothesize that HPV16 E6 contributes to the tumorigenesis and progression of cervical cancer by regulating the expression of host G6PD. Afterwards, the results from the present study obtained *in vitro* verify that hypothesis. With the aid of HPV E6 plasmid transfection, stable HeLa cells that express HPV16 E6 was established. On the basis of the HPV E6 overexpression by pcDNA E6 plasmid, the G6PD overexpression and/or G6PD siRNA interference were also introduced in the HeLa cells to determine whether G6PD was regulated by E6 in the progress of cervical cancer. The data showed that treatment with either E6 or G6PD contributed to the increase in vitality and mobility and decrease in apoptosis of cervical cancer cells.

Previous reports have well established the oncogenic effects of E6 oncoprotein (30) or G6PD (20) in cervical cancer. HPV16 and 18 belong to the high-risk HPV genotype, which is related to more than 70% of cervical cancer and 50% of cervical intraepithelial neoplasia grade 3 (CIN3) (31). HPV16 and/or 18 infection has been proven to be one of the most important pathogenic factors of malignant invasive cervical cancer (1). Currently, eight genes encoded by the HPV genome have been identified, among which E6 and E7 are considered as the major HPV oncoproteins (32). G6PD, a key enzyme in pentose phosphate pathway, has the ability to oxidize glucose 6-phosphate into 6-phosphogluconolactone during the oxidation reaction and simultaneously produce NADPH. In addition, through producing a nucleotide called ribose 5-phosphate, G6PD is involved and essential for cell growth and proliferation. Due to about 85% pentose binding to the DNA derived from the pentose phosphate pathway, G6PD is demonstrated to play important roles in the development and progression of cervical cancer (20). This study demonstrated that E6 played crucial roles in carcinogenic progress, which might be associated with G6PD regulation.

It is interesting that HPV16 E6 regulated the G6PD expression in cervical case and HeLa cells, which might be involved in the carcinogenesis of cervical cancer. Upregulation of HPV16 E6 significantly increased the levels of G6PD transcript and protein. In contrast, interference with HPV16 E6 expression notably reduced G6PD expression. To further verify the reliability of those findings, the G6PD expression upregulated plasmids and/or downregulated siRNAs were conducted into the HeLa cells loading HPV16 E6 overexpressing plasmids (pcDNA E6) and/or E6 siRNA. With the upregulation or downregulation of HPV16 E6 in HeLa cells, G6PD transcript and protein levels correspondingly increased or decreased. Statistical results showed that there was a positive correlation between the expression of HPV16 E6 and G6PD. Furthermore, the proliferation, apoptosis, and mobility of HeLa cells after various plasmids transfection were evaluated. The results revealed that overexpression of HPV16 E6 promoted cell viability, enhanced migration and invasion, and inhibited apoptosis of cervical cancer cells. Downregulation of HPV16 E6 reduced cell viability, lessened migration and invasion, and promoted apoptosis of cervical cancer cells. Combining with G6PD siRNA and pcDNA E6 treatments attenuated those effects induced by pcDNA E6, while upregulation of G6PD and E6 enhanced those effects induced by overexpression of HPV16 E6. Simultaneously, transfection with G6PD overexpression plasmids restored the vitality and mobility, and inhibited apoptosis of HPV16 E6 siRNA-treated HeLa cells. Treatment with both siRNA of HPV16 E6 and G6PD significantly inhibited the viability, migration, and invasion, as well as greatly increased the apoptosis rate of tumor cells. Moreover, luciferase reporter assay and ChIP assay verified the transcriptional regulation of HPV16 E6 by directly binding to the promoter of G6PD. Therefore, these results suggested that HPV16 E6 was involved in the development of cervical cancer by regulating the expression of host G6PD. The E6 and E7 oncoproteins can bind to and stimulate the degradation of the tumor suppressors p53 (33, 34) and pRb (35), which are essential for cell cycle control. It is reported that E6 protein is involved in complex regulatory patterns of gene expression in the host cells induced by HPV infection, which disturbs host miRNA expression and is related to tumorigenesis (24). Others revealed that HPV16 E6 inhibits p53 and thereby suppresses the apoptosis of tumor cells (36), which plays important roles in cancer cell survival and tumor progression (37).

Given those, the results revealed that HPV16 E6 had a function to act as a regulatory factor and alter the viability and mobility of cervical cancer cells by regulating the expression of G6PD, which eventually induced the progression and metastasis of cervical cancer. Furthermore, this study found that HPV16 E6 activates the transcription of G6PD and directly binds to the promoter of G6PD. However, the potential mechanism of the interaction between E6 and G6PD and the initial activating G6PD transcription by E6 are still unclear, which needs to be further studied by future researchers. The results of this study provide a solid foundation for further exploring the relationship between HPV16 E6 and G6PD in the carcinogenesis of cervical cancer, and supply novel targets for diagnosis and therapy.

## Data Availability Statement

The raw data supporting the conclusions of this article will be made available by the authors, without undue reservation.

## Ethics Statement

The studies involving human participants were reviewed and approved by the Human Ethics Committee and the Research Ethics Committee of Kunming University of China. The patients/participants provided their written informed consent to participate in this study.

## Author Contributions

All authors contributed to the study conception and design. Y-FC, G-CL, YH, Y-BX, and TH conceptualized the idea of this article and proposed the hypothesis. Y-FC, G-JY, G-CL, YH, H-LC, SJ, and YZ performed the material preparation, experiments, and data collection. G-JY, SJ, and Y-BX performed formal analysis and investigation. G-JY analyzed the data and wrote the first draft of the manuscript. Y-BX and TH reviewed and edited the manuscript. All authors contributed to the article and approved the submitted version.

## Funding

This work was supported by the Medical Reserve Talents Cultivation Project of the Health and Family Planning Commission of Yunnan Province (Grant No. H-2017026).

## Conflict of Interest

The authors declare that the research was conducted in the absence of any commercial or financial relationships that could be construed as a potential conflict of interest.

## Publisher’s Note

All claims expressed in this article are solely those of the authors and do not necessarily represent those of their affiliated organizations, or those of the publisher, the editors and the reviewers. Any product that may be evaluated in this article, or claim that may be made by its manufacturer, is not guaranteed or endorsed by the publisher.
